# Comparing the Efficacy of Intra-Articular Application of Morphine and Tramadol on Postoperative Pain After Arthroscopic Knee Surgery

**DOI:** 10.5812/aapm.4815

**Published:** 2012-07-10

**Authors:** Seyed Mohammad Jazayeri, Faramarz Mosaffa, Mohammadreza Abbasian, Hamid Reza Hosseinzadeh

**Affiliations:** 1 Akhtar Orthopedic Hospital, Shahid Beheshti University of Medical Sciences, Tehran, Iran; 2 Department of Anesthesiology, Akhtar Orthopedic Hospital, Shahid Beheshti University of Medical Sciences, Tehran, Iran

**Keywords:** Arthroscopy, Knee Injuries, Pain, Postoperative, Morphine, Tramadol

## Abstract

**Background:**

Intra-articular analgesia is a pain reliever that is frequently administered following arthroscopic knee surgery.

**Objectives:**

The purpose of this study was to compare the efficacy of intra-articular application of morphine and tramadol on postoperative pain after arthroscopic knee surgery.

**Patients and Methods:**

For this randomized double blinded clinical trial, 132 patients undergoing minor arthroscopic knee surgery were randomly assigned to receive either; 5 mg morphine or 50 mg tramadol intra-articularly. Pain was evaluated by means of the verbal pain rating score (VRS) preoperatively (at rest and on movement of the knee joint) and postoperatively at 0, 1, 2, 3, 4, 6, 12 and 24 hours. Meanwhile, the time of the first analgesic request and need for supplemental analgesic were also recorded.

**Results:**

There was no statistically significant difference in VRS scoring between the two groups during the preoperative period either at rest or on knee movement. Meanwhile, VRS scores did not differ significantly between the morphine and tramadol treated groups postoperatively, except for in the one-hour post-operative scores in which the tramadol-treated group experienced less pain (*P* < 0.007). Post-operative VRS scores at 6, 12, and 24 hours were significantly decreased when compared with previous scores in both morphine and tramadol prescribed subjects (*P* < 0.001), hence, both local analgesics can significantly reduce pain after minor knee surgery.

**Conclusions:**

We have found a postoperative analgesic effect of intra-articularly administered morphine and tramadol following minor arthroscopic knee surgeries with a maximum effect 6 hours post injection.

## 1. Background

Arthroscopic surgery of the knee is frequently undertaken as either a diagnostic or therapeutic approach for knee problems. Postoperatively, different techniques have been applied for pain relief such as non-steroidal anti-inflammatory drugs, intra-muscular and intravenous opioids, intrathecal and epidural narcotics, and patient-controlled analgesia, most of which are associated with side effects (1, 2). Intra-articular local anesthetics such as bupivacaine provide post-operative analgesia of immediate onset, but short duration, following which patients require supplementary analgesia (3). Nevertheless, the intra-articular administration of narcotic-like local analgesics has received further attention during the past decades. Opiates such as morphine and tramadol have peripheral and central analgesic effects, and there is evidence that there are opiate receptors at the terminals of afferent peripheral nerves, therefore, administration of opiates peripherally might provide a significant analgesic effect (4, 5). Gramsch et al. reported the presence of local opioid receptors in peripheral inflamed tissues (6). This peripheral effect of narcotic-like analgesics could explain why the intra-articular administration of morphine and tramadol could provide a satisfactory pain-relief state as well as fewer systemic side effects.

## 2. Objectives

The present study aimed to evaluate the efficacy of intra-articular application of morphine and tramadol on postoperative pain after arthroscopic knee surgery.

## 3. Patients and Methods

For this randomized double blinded clinical trial, 132 patients undergoing minor arthroscopic knee surgery under general anesthesia were enrolled. We included patients who had undergone; partial meniscectomy, chondroplasty, removal of loose bodies and diagnostic arthroscopy. Patients were randomly assigned to receive 5 mg morphine or 50 mg tramadol intra-articularly. All solutions were injected at the end of the operation and before tourniquet release, with the tourniquet being released 10 minutes after the injection was administrated. Preoperatively, patients were instructed to report their pain with a verbal pain rating score (VRS) at rest and on movement of the knee joint. Postoperative pain was also assessed by VRS at 0, 1, 2, 3, 4, 6, 12 and 24 hours. VRS is a 10-point scale tool for evaluation of pain intensity; “0” represents “no pain,” while “10” is the “worst imaginable pain”. Furthermore, the patients were monitored post-operatively for a period of 24 hours for the following parameters; pulse rate, blood pressure, respiratory rate, level of analgesia, time of first analgesic request (usually associated with VRS > 3) and need for supplemental analgesic. Supplemental analgesia was provided with diclofenac sodium oral tablets and total consumption was recorded over 24 hours. Meanwhile, the following exclusion criteria were applied at baseline; evidence of any major systemic illness, major knee surgery, history of allergy to any of the drugs in the study, morphine addiction, regional anesthesia or history of convulsive disorders.

The study protocol was approved by the Ethics Committee of university and all patients were requested to give their informed consent. Results are expressed as mean ± standard deviation (SD) for continuous variables, unless otherwise stated. VRS results were analyzed by a Mann-Whitney U test, however, other data were assessed using a Student t-test and chi-square test, when appropriate. For all tests, significance was defined as *P* < 0.05. All statistical analyses were achieved using SPSS software (SPSS version 13.0, SPSS Inc., Chicago, USA).

## 4. Results

The study population included; 53 males and 22 females in the morphine and 39 males and 18 females in the tramadol group with a mean age of 41.0 ± 11.6 and 40.5 ± 10.9 years, respectively. In total, 80% of the morphine vs. 84.2% of the tramadol-treated subjects received midazolam plus fentanyl for general anesthesia, however, the difference did not reach a statistically significant level. Table 1 summarizes the demographic and clinical features of patients in both groups. None of the measured features differed significantly between the morphine and tramadol groups. The time of the first analgesic request and the first postoperative pain report did not differ significantly between the two groups. Table 2 compares pre- and postoperative VRS scores. There was no statistically significant difference in VRS scoring between the two groups during the preoperative period either at rest or on knee movement. Results also revealed that VRS scores did not differ significantly between the morphine and tramadol treated groups postoperatively, except for the one hour post-operative scores in which the tramadol-treated group experienced less pain (P < 0.007). Meanwhile, as shown in Figure, post-operative VRS scores at 6, 12, and 24 hours were significantly decreased when compared with previous scores in both morphine and tramadol prescribed subjects (P < 0.001), hence, both local analgesics can significantly reduce pain after minor knee surgery through a local opioid receptor-specific site of action, reaching a maximum effect 6 hours post-injection. Finally, there was no statistically significant difference in the total consumption of supplemental analgesia between the two groups.

**Table 1:** Demographic and Clinical Features of Patients Undergoing Minor Knee Arthroscopic Surgeries According to the Prescribed Analgesic

	**Morphine Group, n = 75**	**Tramadol Group, n = 57**	***P* value**
Age, y, mean ± SD	41.0 ± 11.6	40.5 ± 10.9	0.89
Gender, No. (%)			
Female	22 (29.3)	18 (31.6)	0.51
Male	53 (70.7)	39 (68.4)	
Duration of chief complaint, mo, mean ± SD	25 ± 9	27 ± 14	0.68
Type of analgesic, No. (%)			
Midazolam	15 (20)	9 (15.8)	0.42
Midazolam + fentanyl	60 (80)	48 (84.2)	
Time to first analgesic request, h, mean ± SD	2.2 ± 2.0	2.7 ± 1.7	0.27
Time to first postoperative pain report, h, mean ± SD	1.5 ± 1.1	1.2 ± 0.8	0.33
Duration of operation, h, mean ± SD	2.1 ± 0.5	1.8 ± 0.7	0.11

**Table 2:** Pre-and Post-Operative Verbal Pain Rating Score (VRS) at 0, 1, 2, 3, 4, 6, 12 and 24 Hours of Patients Undergoing Minor Knee Arthroscopic Surgeries According to the Prescribed Analgesic

**Time of VRS Checking**	**Morphine Group, n = 75**	**Tramadol Group, n = 57**	***P* value**
Pre-operative, mean ± SD			0.27
At rest	5.0 ± 2.3	4.6 ± 2.5	0.89
On movement of the knee joint	6.1 ± 2.5	5.9 ± 2.6	
Post-operative, h, mean ± SD			
0	5.6 ± 3.7	5.2 ± 1.8	0.71
1	6.3 ± 3.4	4.9 ± 3.4	0.007
2	4.5 ± 2.9	5.0 ± 3.3	0.46
3	4.1 ± 2.4	4.7 ± 3.0	0.21
4	3.8 ± 2.5	3.6 ± 2.6	0.52
6	3.3 ± 2.8 ^a^	3.6 ± 2.7 ^a^	0.99
12	2.2 ± 1.5 ^a^	2.8 ± 2.1 ^a^	0.21
24	2.0 ± 1.4 ^a^	2.3 ± 1.6 ^a^	0.24

a P < 0.001 when compared with previous VRS scores

**Figure:**
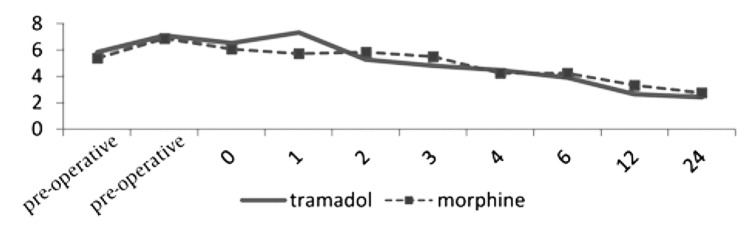
Pain Intensity According to Verbal Pain Rating Score (VRS) in Morphine-and Tramadol-Treated Group

## 5. Discussion

Knee arthroscopy is a common day-surgery procedure for which effective pain relief is of the utmost importance, not only for same day discharge, but also to improve comfort and mobility at home (2, 7). In the present study, we have sought to evaluate the efficacy of intra-articularly administered morphine and tramadol on postoperative pain following minor arthroscopic knee surgery. Our results revealed improvements in postoperative pain relief, with both local analgesics. Stein et al. showed that low doses of intra-articular morphine, injected on the completion of arthroscopic knee surgery can produce post-operative analgesia via the activation of local opioid receptors in the knee joint (8). Several other studies have shown similar findings, however, Heard found no benefit with morphine compared to controls (9). Other studies have proposed different intra-articular solutions as post-operative pain relievers. Injection of bupivacaine following arthroscopy has been demonstrated to be both safe and effective in providing post-operative analgesia, although the mean duration of analgesia is only two hours (10). Moreover, intra-articular clonidine has been reported to provide post-operative analgesia for up to 533 minutes in patients undergoing arthroscopic knee surgery under regional anesthesia (11), unfortunately, both of these drugs are associated with systemic side effects. In our study, morphine-treated patients reported postoperative pain on average 1.5 hours following their operation and requested supplemental analgesics on average 2.2 hours postoperatively. In contrast, tramadol-treated subjects reported pain at 1.2 hours and requested supplemental analgesics 2.7 hours postoperatively. Hence, tramadol is associated with shorter, but relatively more satisfactory pain-relief in comparison with morphine. Nonetheless, a shorter duration of operation (1.8 vs. 2.1 hours), lower preoperative VRS scores (4.6 and 5.9 vs. 5.0 and 6.1 at rest and on movement of knee joint, respectively), and smaller sample size (57 vs. 75 subjects) could misinterpret this conclusion. Furthermore, post-operative VRS scores at 6, 12, and 24 hours were significantly decreased when compared with previous scores in both morphine and tramadol prescribed subjects. Therefore, we can conclude that both local analgesics reach a maximum effect six hours post-injection and their effect lasts for at least 18 hours. In contrast, in a study by Iqbala et al., six patients in the control group received rescue analgesic in the first postoperative hour compared to none in the morphine group. They concluded that the onset of intra-articular morphine action is immediate and intra-articular morphine provided significant post-operative analgesia for three hours with the highest rescue analgesic requirement in the fourth post-operative hour (1). Disparities between the different studies could be explained in part by the varied doses of local anesthesia used in the intra-articular instillation. Of more importance may be the VRS system of pain scoring that is patient-dependent and is relatively lacking in sensitivity, especially when pain is absent or even mild.

Our study had some limitations. Lack of a control group receiving a placebo is the major drawback. Like other studies of the same protocol, VRS is an insensitive scoring system. In conclusion, based on the data presented in this study, we have found a postoperative analgesic effect of intra-articularly administered local anesthesia following minor arthroscopic knee surgery with a maximum effect 6 hours post injection.
